# Supplementary material for: Hybrid Structures of Sisal Fiber Derived Interconnected Carbon Nanosheets/MoS2/Polyaniline as Advanced Electrode Materials in Lithium-Ion Batteries
Source: Molecules. 2021 Jun 18;26(12):3710. doi: 10.3390/molecules26123710 (PMC8233771; doi:10.3390/molecules26123710)
Supplement: Supplementary file 1 [file molecules-26-03710-s001.zip › molecules-1169252-supplementary.pdf]

Electronic supporting information

Caramel treats-like sisal fiber derived interconnected carbon nanosheets/MoS_2/polyaniline hybrid as advanced electrode materials in lithium-ion batteries

Yuanzhou Liu[1], Lixue Wei[1], Lei Liao*[2], Kaiyou Zhang[1], Shuoping Chen[1], Aimiao Qin*[1]

[1]Key Lab New Processing Technology for Nonferrous Metals & Materials Ministry of Education, Guangxi Key Laboratory in Universities of Clean Metallurgy and Comprehensive Utilization for Non-ferrous Metals Resources, College of Materials science & engineering, Guilin University of Technology, Guilin, China

[2]Guangxi Key Laboratory of Environment Pollution Control Theory and Technology, Co llege of Environmental Science and Engineering, Guilin University of Technology Guilin, China

*Corresponding Authors

Email: 592491245@qq.com (Aimiao Qin)

Email: fangqiu2001@163.com(Lei Liao)

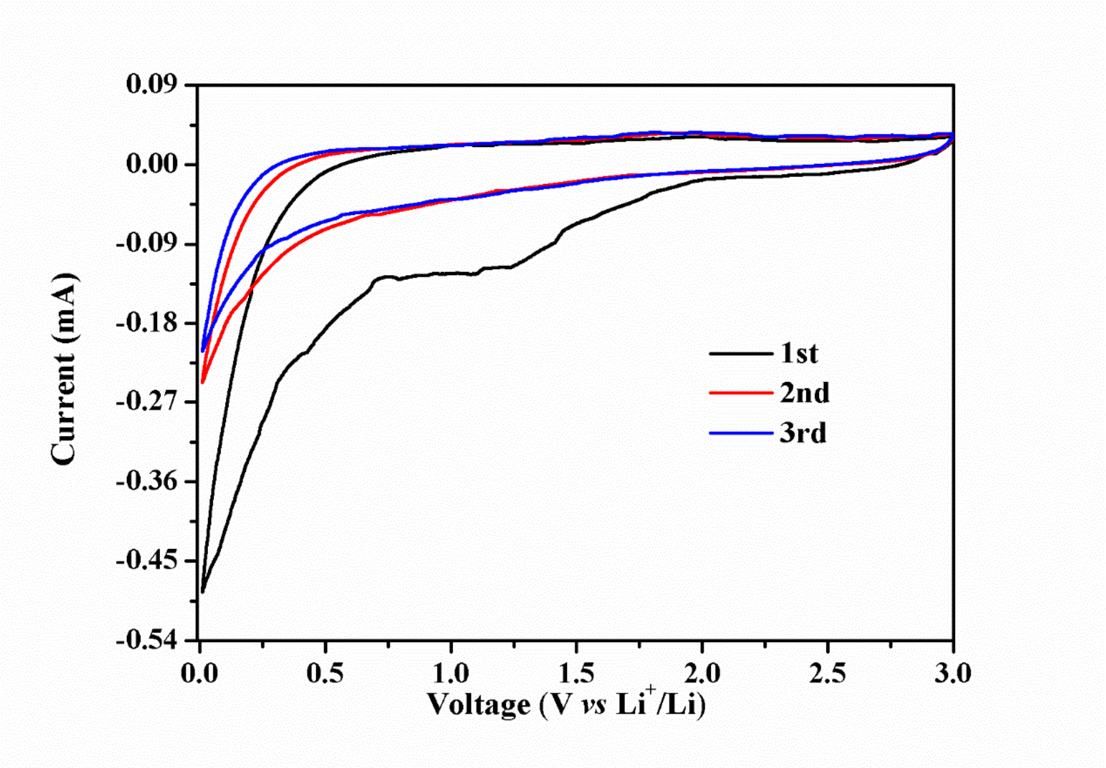

Figure S1 Cyclic voltammograms of the ICNs/PANI electrodes at a scan rate of 0.2 mV/s

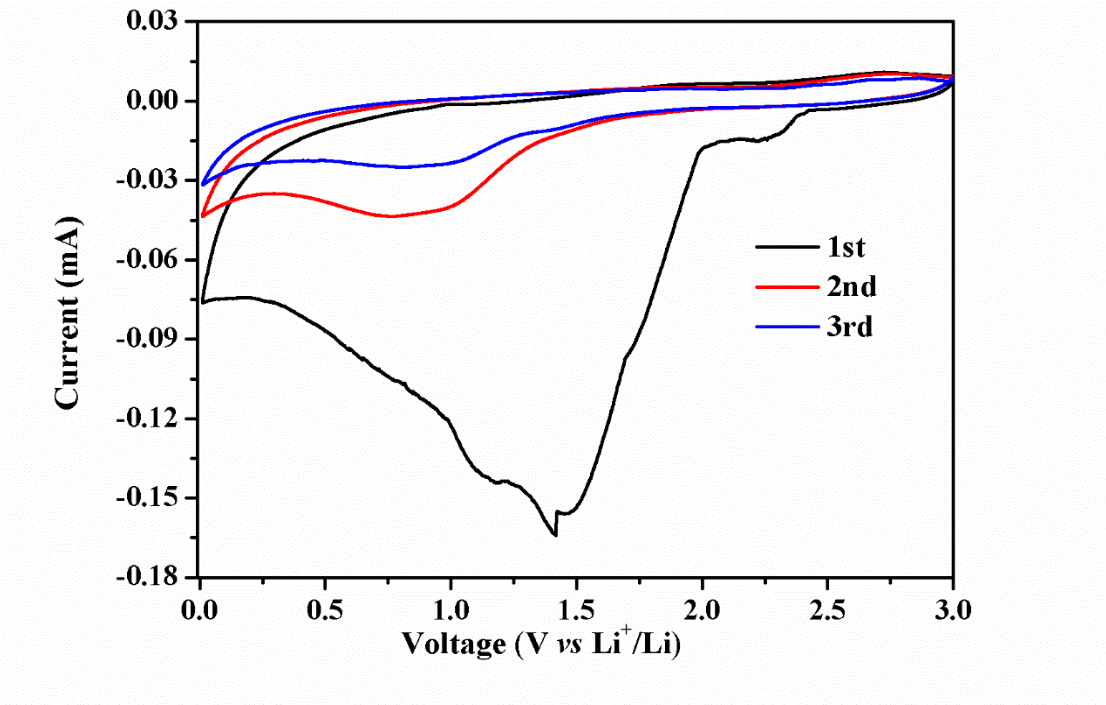

Figure S2 Cyclic voltammograms of the PANI electrodes at a scan rate of 0.2 mV/s

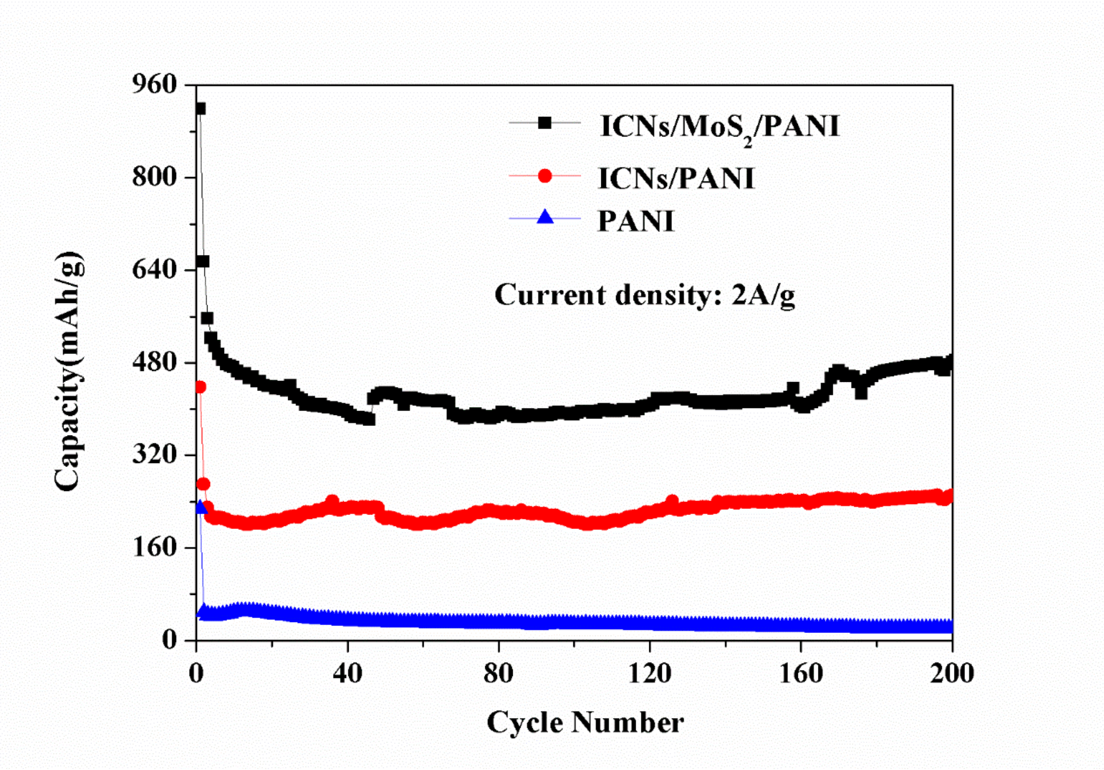

Figure S3 Cycling performance of the ICNs/MoS_2/PANI hybrid, ICNs/PANI and PANI at a current density of 2A/g for 200 cycles

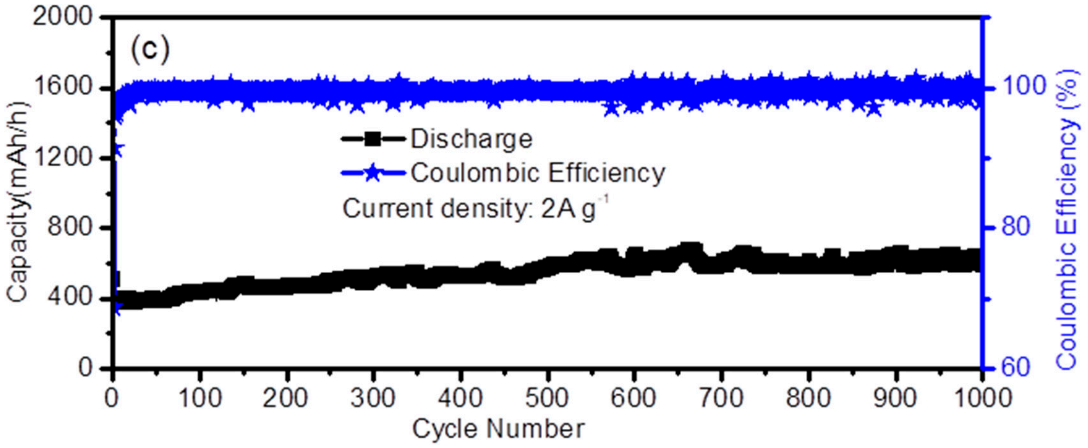

Figure S4 Cycling performance and Coloumbic efficiency of the ICNs/MoS_2/PANI hybrid at a current density of 2A/g for 1000 cycles

The preparation of interconnected carbon nanosheets(ICNs)：

The sisal fibers derived ICNs were prepared through two stages, which illustrated in Figure S5.

First, we prepared tubular cell-fibers from sisal fibers (SFs). The SFs were supplied by Guangxi Sisal Group Co., Ltd. The maize-yellow SFs were washed with deionized water to remove the impurities, cut into small pieces (~2cm in length) and dried in the oven at 80°C. And then the SFs were treated by hydrothermal reaction. 5.0g of SFs, 70mL of 2.5M potassium hydroxide (KOH) solution were placed in a 100-mL Teflon-lined stainless steel autoclave. The mixture solution was heated at 160°C for 14h, and cooled to room temperature. The tawny prepared materials were collected and washed by deionized water until the pH decreased to 7, dried in the oven at 80°C, and the tubular cell-fibers can be obtained. The morphological structure of the ICNs is shown in Figure S6.

Second, we prepared the ICNs from tubular cell-fibers. The tubular cell-fibers were immersed in KOH solutions, in which the weight ratio of KOH to tubular cell-fibers is 2.5. The solution was stirred for 30 min, and dried in an oven at 70°C. Then the dry mixture was placed in a nickel boat for carbonization at 400°C for 2 h, and for activation at 850°C for 1h. The heating rate going from room temperature to 400 and 850°C was 3°C min^{-1} under nitrogen atmosphere. The resulting sample was collected,

grinded fully, and thoroughly washed with 3 M hydrochloric acid (HCl) solution, and then washed with deinoized water until the pH decreased to 7. The samples were finally dried at 60°C in the oven, and the ICNs were prepared completely.

Preparation of PANI:

PANI is prepared by in-situ oxidative polymerization of aniline monomer using ammonium persulfate ($(NH_4)_2S_2O_8$, APS) as an oxidizing agent. Usually, 2 mL of aniline is added to 70 mL of 1.5 M H_2SO_4 under vigorous magnetic stirring, stirred vigorously for 2 h, and then a molar ratio of APS is used to add a ratio of 1:0.5 (aniline: APS) to the solution, and at room temperature The reaction was continued for another 12 hours while stirring.

Preparation of ICNs/PANI:

This hybrid was prepared by in situ oxidative polymerization of aniline monomers on ICNs/PANI using ammonium persulfate ($(NH_4)_2S_2O_8$, APS) as oxidizer. Typically, 50 mg of precursor ICNs and 70 mL of 1.5 M H_2SO_4 were added in a flask with vigorous magnetic stirring for 15 min, and then 0.045 mL aniline was injected into the solution, continued vigorous stirring for 2 h, then APS with a molar ratio of 1:0.5 (aniline: APS) was added into the solution, and reacting for another 12 h under constant stirring at room temperature.

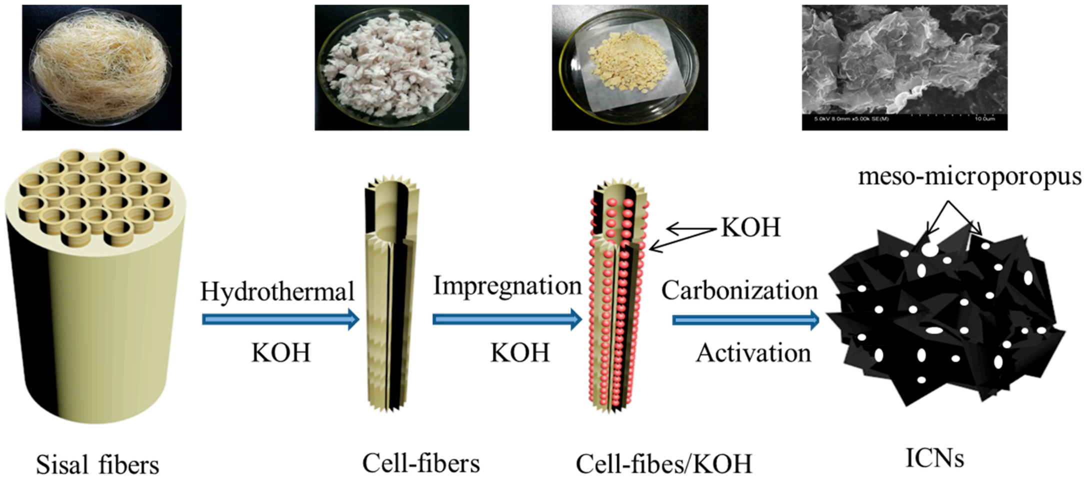

Figure S5 The Schematic illustration of the synthesis procedure of ICNs

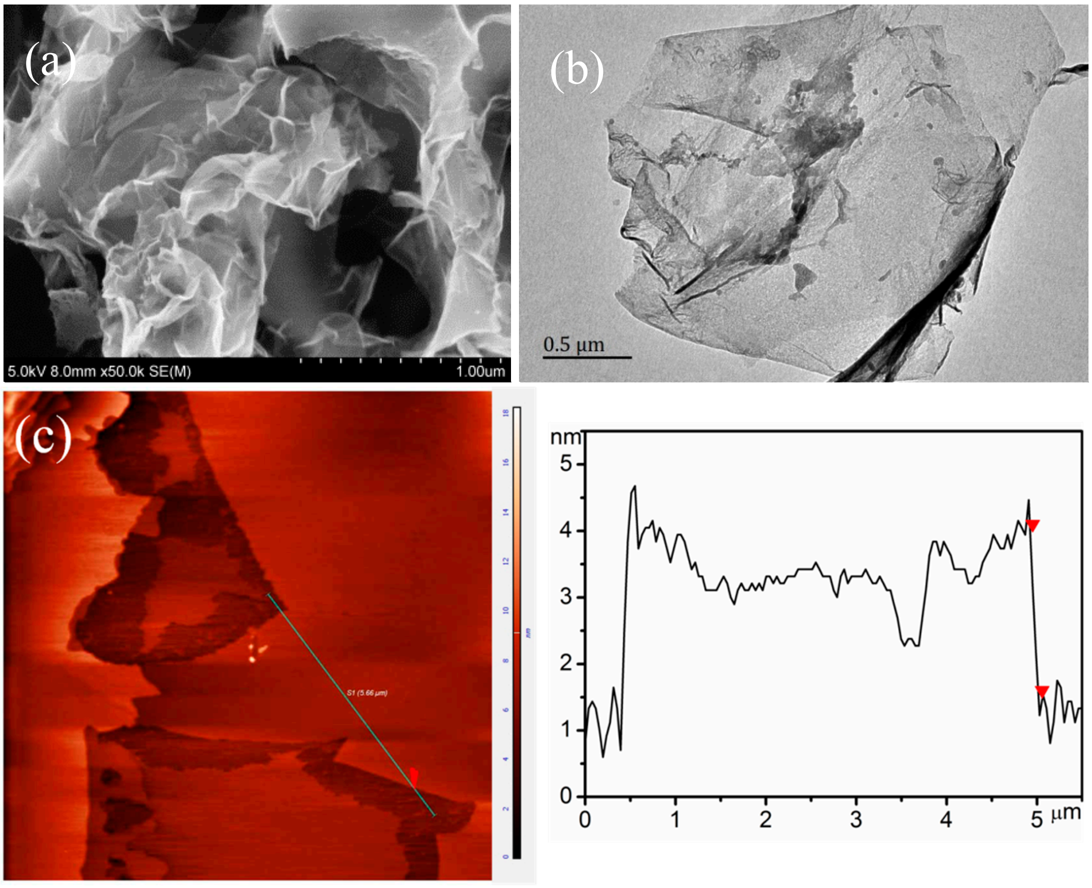

Figure S6 (a) SEM (b) TEM and (c) AFM images of ICNs
